# Factors Involved in the Progression of Preclinical Atherosclerosis in People with Type 1 Diabetes

**DOI:** 10.3390/jcm14176004

**Published:** 2025-08-25

**Authors:** Clara Viñals, Ignacio Conget, Montse Granados, Clara Solà, Denisse Ayala, Alex Mesa, Tonet Serés-Noriega, Mònica Domenech, Verónica Perea, Jesús Blanco, Irene Vinagre, Marga Giménez, Antonio J. Amor

**Affiliations:** 1 Diabetes Unit, Endocrinology and Nutrition Department, Hospital Clínic, Villarroel 170, 08036 Barcelona, Spain; 2 The Clinic Foundation for Biomedical Research, August Pi Sunyer Biomedical Research Institute (IDIBAPS), 08036 Barcelona, Spain; 3 Diabetes and Associated Metabolic Diseases Networking Biomedical Research Centre (CIBERDEM), 28029 Madrid, Spain; 4 Endocrinology and Nutrition Department, Hospital de la Santa Creu i Sant Pau, 08025 Barcelona, Spain; 5 Endocrinology and Nutrition, Centro Médico Milenium, 50006 Zaragoza, Spain; 6 Endocrinology and Nutrition Department, Hospital Mutua Terrassa, 08221 Terrassa, Spain

**Keywords:** atherosclerosis, cardiovascular risk factors, carotid atherosclerosis, carotid ultrasound, Steno Type 1 Risk Engine, type 1 diabetes

## Abstract

**Background/objectives:** Individuals with type 1 diabetes (T1DM) face an elevated risk of cardiovascular disease (CVD), yet the factors driving atherosclerosis remain unclear. This study aimed to assess factors associated with preclinical atherosclerosis development or progression in T1DM. **Methods:** We conducted a prospective study in T1DM individuals without established CVD, aged ≥40 years, with diabetic kidney disease and/or ≥10 years of T1DM plus another cardiovascular risk factor (CVRF). Baseline evaluation followed a standardized CV risk assessment protocol, including carotid ultrasound and cardiovascular risk estimation using the Steno Type 1 Risk Engine (ST1RE). Ultrasound was repeated after 3–5 years; progression was defined as an increase in plaque number. CVRF control was considered optimal when LDL-cholesterol was within target based on atherosclerotic burden, blood pressure <130/80 mmHg, HbA1c <7%, and non-smoking status. Logistic regression models identified predictors of progression. **Results:** We included 151 participants (55.6% women; mean age 49.8 ± 8.9 years; T1DM duration 27.3 ± 9.1 years); 42.4% had plaques at baseline. Over a follow-up of 5.22 ± 1.29 years, despite improved CVRF control (*p* < 0.05), 40.4% experienced progression. Older age (OR 1.38 [1.1–1.8]) and active smoking (OR 3.29 [1.4–7.5]) were significant predictors of progression. Baseline cardiovascular risk measured by the ST1RE independently predicted progression (OR 1.09 [1.03–1.15]) after adjusting for other CVRFs. Persistent smoking (OR 2.52 [1.06–5.99]) and baseline ST1RE (OR 1.06 [1.02–1.11]) remained significant after accounting for baseline and follow-up CVRFs. **Conclusions:** Despite improved CVRF control, atherosclerosis progression is common in T1D. ST1RE may help identify individuals at highest risk for targeted preventive strategies.

## 1. Introduction

People living with Type 1 diabetes mellitus (T1DM) are more prone to develop cardiovascular disease (CVD), which remains the leading cause of morbidity and mortality [1]. Life expectancy among individuals with T1DM is approximately 13 years shorter than that of the general population, with the greatest impact observed in those diagnosed at an early age [2,3]. Cardiovascular risk (CVR) escalates with each uncontrolled risk factor, even when glycemic control is adequate, highlighting the need for comprehensive CVD risk management [4,5].

The pathophysiology of CVD in T1DM is multifactorial, involving both traditional and diabetes-specific risk factors [6,7]. Non-invasive imaging methods like carotid ultrasound can identify atherosclerosis, a systemic condition that precedes CVD. The development of atheromatous plaques has been associated with future cardiovascular events, as shown in large population studies without diabetes [8,9]. Recently, subclinical atherosclerosis has been linked to increased mortality and major cardiovascular events in T1DM [10]. Furthermore, the progression of atherosclerosis is linked to cardiovascular events [11] and mortality [12] in the general population. Importantly, plaque progression is a controllable phase in the illness trajectory [13]. Identifying the factors that drive atherosclerosis progression is essential for developing treatments to slow or prevent its development.

There are few investigations on the evolution of subclinical atherosclerosis in the general population [14], and such research is especially scarce in T1DM.

Given this background, our study aimed to identify factors associated with the development and progression of preclinical atherosclerosis in individuals with T1DM.

## 2. Materials and Methods

### 2.1. Study Participants

This was a prospective study based on a structured protocol for CVR assessment in individuals with T1DM attending the specialized diabetes unit at a tertiary referral hospital in Catalonia, Spain. The protocol included a systematic evaluation of cardiovascular risk factors (CVRFs) and standardized carotid ultrasonography [15,16,17]. Participants were eligible if they had no history of CVD and met at least one of the following criteria: age ≥40 years, presence of diabetic kidney disease (DKD), or T1DM duration ≥10 years with an additional CVRF. Additional CVRFs included diabetic retinopathy (DR), LDL cholesterol (LDL-C) >160 mg/dL or statin use, low HDL cholesterol (<40/45 mg/dL in men/women), triglycerides ≥150 mg/dL, hypertension, premature CVD in first-degree relatives (<55/65 years in men/women), active smoking, history of eclampsia/preeclampsia, hypoglycemia unawareness (Clarke test >3) [18], or a prior episode of severe hypoglycemia. We included this high-risk population based on statin treatment criteria outlined in current clinical guidelines [19].

All participants provided written informed consent, and the study adhered to the Declaration of Helsinki.

### 2.2. Clinical and Laboratory Measures

Demographic and clinical data were collected according to established protocol [17]. Laboratory tests included serum creatinine, urinary albumin-to-creatinine ratio (ACR), HbA1c, fasting glucose, and lipid profiles (total cholesterol, triglycerides, and HDL cholesterol by direct methods; LDL cholesterol by the Friedewald formula). Estimated glomerular filtration rate (eGFR) was calculated using the CKD-EPI equation, while HbA1c was analyzed centrally via high-performance liquid chromatography and reported using NGSP/DCCT standards. Anthropometric and blood pressure (BP) measurements were obtained using standardized procedures.

Diabetic kidney disease (DKD) was defined as ACR ≥ 30 mg/g (confirmed in ≥2 of 3 samples) and/or eGFR < 60 mL/min/1.73 m^2^; use of ACEi/ARBs in the absence of hypertension or CVD was also considered indicative of DKD. Diabetic retinopathy (DR) was diagnosed by ophthalmologic fundus examination. Hypertension was defined by systolic BP ≥140 mmHg, diastolic BP ≥ 90 mmHg on repeated measurements, or use of antihypertensive therapy (excluding ACEi/ARB use for DKD), in line with contemporaneous guidelines [20].

Cardiovascular risk was estimated using the Steno Type 1 Risk Engine (ST1RE), incorporating 10 clinical variables [21]. Insulin sensitivity was assessed using the estimated glucose disposal rate (eGDR) formula [22,23]. The eGDR was calculated as follows: 24.31–12.22 × waist-to-hip ratio − 3.29 × hypertension (0 = no, 1= yes) − 0.57 × HbA1c (in %).

### 2.3. Carotid B Ultrasound Imaging

All participants underwent bilateral carotid artery ultrasounds using high-resolution B-mode imaging with 5–10 MHz transducers (ACUSON X700 [Siemens Healthineers] or Aplio a450 [Canon]). Both were available and operational throughout the entire study period, with identical settings and transducers applied at baseline and follow-up assessments. Carotid imaging followed predefined protocols for the evaluation of intima–media thickness (IMT) and plaque detection [16,17,24]. All examinations were conducted exclusively by two endocrinologists with extensive expertise in vascular ultrasound (AJA and CV), ensuring standardized acquisition and measurement procedures.

Carotid ultrasound follow-up intervals were based on baseline findings. Re-evaluation occurred every 5 years in patients with non-severe or absent preclinical atherosclerosis (two or fewer plaques < 3 mm height and no significant stenosis). Re-evaluation every 3 years was conducted in the presence of findings indicating severe preclinical atherosclerosis (three or more plaques or one plaque of at least 3 mm height). An annual examination was performed on patients with significant stenosis (≥50%). Progression was defined as an increase, and regression as a decrease, in plaque number between evaluations.

### 2.4. Cardiovascular Risk Assessment, Attainment of Cardiovascular Risk Factor Goals, and Follow-Up

As mentioned before, the study was conducted following the protocol published elsewhere [17] and was used to guide cardioprotective treatment. We assessed the achievement of CVRF targets according to current clinical guidelines [17,25,26,27]. Specifically, we considered the attainment of HbA1c < 53 mmol/mol (<7%), BP < 130/80 mmHg, and LDL-C levels < 100 mg/dL in individuals without plaques, <70 mg/dL in those with fewer than 3 plaques and plaques < 3 mm in height, and <55 mg/dL in those with ≥3 plaques or with any plaque ≥ 3 mm in height.

In this study, two assessments were conducted for each participant: an initial assessment and a follow-up assessment, guided by carotid ultrasound following the criteria established in the previous section.

### 2.5. Statistical Analysis

Data are presented as mean ± standard deviation (SD) for normally distributed continuous variables, median with interquartile ranges for non-normally distributed continuous variables, or as numbers with percentages for categorical variables. Normality was assessed using the Kolmogorov–Smirnov test. Comparisons by progression status, estimated cardiovascular risk by ST1RE, plaque presence, and sex were conducted using unpaired Student’s *t*-tests or Mann–Whitney U tests for continuous variables and chi-squared or Fisher’s exact tests for categorical variables.

Logistic regression models were used to identify predictors of progression. Model 1 included the ten components of the ST1RE score; Model 2, the categorized ST1RE score; and Model 3, the ST1RE score plus additional cardiovascular risk factors (CVRFs) not included in the score (e.g., carotid plaques, DR, eGDR, non-HDL-C, BMI, statin use, severe hypoglycemia). All models were adjusted for follow-up duration. An exploratory analysis in women assessed the impact of preeclampsia in Model 3.

We examined changes between initial and final evaluations concerning statin treatment, LDL-C, hypertension, SBP, smoking status, and BMI relative to initial ST1RE score, plaque presence, progression status, and sex. Paired binomial proportions were compared using the McNemar test, while the Friedman test was used to analyze paired non-dichotomous proportions. Continuous variables were analyzed using the paired *t*-test. Changes in CVRFs (BMI, SBP, LDL cholesterol, smoking status, and statin treatment) were incorporated alongside plaque presence, the ST1RE score, and its change in logistic regression models to identify predictors of plaque progression.

Finally, the achievement of optimal cardiovascular risk factors is in accordance with current clinical guidelines [17,25,26,27]. LDL-C levels based on plaque presence and burden, (BP < 130/80 mmHg and HbA1c < 53 mmol/mol (<7%)) were evaluated based on the ST1RE score, plaque presence, progression status, and sex. Comparisons of proportions between groups were conducted using the chi-squared test or Fisher’s exact test, as appropriate. Paired binomial proportions were analyzed with the McNemar test, and paired non-dichotomous proportions were assessed using the Friedman test.

All statistical analyses were conducted using IBM SPSS Statistics version 29.0 (SPSS Inc., Chicago, IL, USA). A two-tailed *p*-value < 0.05 was considered statistically significant.

## 3. Results

### 3.1. Baseline Characteristics Associated with Atherosclerosis Progression

We included 151 participants (55.6% female, mean age of 49.8 ± 8.9 years, and 27.3 ± 9.1 years of diabetes duration). At baseline, the prevalence of microvascular complications (both DR and DKD) was 47.4% (*n* = 72), and 42.2% (*n* = 64) harbored at least one carotid plaque. The mean follow-up time was 5.22 ± 1.29 years.

**Figure 1:**
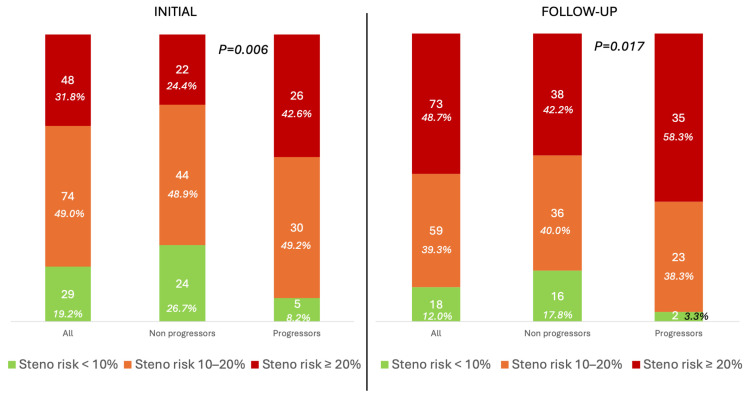
Steno Type 1 Risk Engine according to progression status. The data are presented as number and percentage.

During the follow-up period, 40.4% (*n* = 61) of the participants showed progression in the number of carotid plaques, while regression was observed in only 3.3% (*n* = 5). The clinical characteristics, laboratory data, and treatment of the study population stratified by progression status are presented in Table 1. Progressors were older and more likely to be active smokers, with higher cumulative tobacco consumption, compared to non-progressors (*p* < 0.05 for all comparisons). The overall plaque presence did not differ significantly between groups when categorizing participants by no plaques, non-severe atherosclerosis (defined as <3 plaques with <3 mm height), or severe atherosclerosis (defined as ≥3 plaques or one plaque ≥3 mm in height; *p* > 0.05). However, progressors exhibited a greater plaque burden, evidenced by a higher presence of ≥2 and ≥3 plaques (see Table 1; *p* < 0.05 for both). Regarding CVR assessed by the ST1RE score, progressors had an estimated higher risk than non-progressors, with 42.6% of progressors having an ST1RE score of ≥20% compared to 24.4% of non-progressors (*p* < 0.001; see Figure 1 and Table 1).

New-onset atherosclerosis accounted for half of the individuals who showed disease progression (*n =* 31; 50.81%).

### 3.2. Baseline Predictors of Atherosclerosis Progression

**Figure 2:**
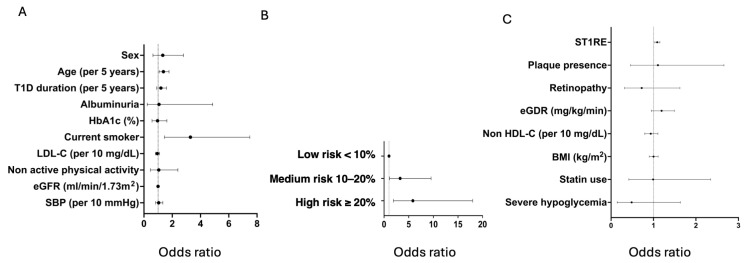
Logistic regression models with factors associated with the progression of atherosclerosis. (**A**): With the individual ST1RE variables; (**B**): With the ST1RE category. Low risk as reference; (**C**): With the ST1RE score plus other cardiovascular risk factors. Abbreviations: BMI: body mass index; C: cholesterol; eGDR: estimated glucose disposal rate; eGFR: estimated glomerular filtration rate; SBP: systolic blood pressure; ST1RE: Steno Type 1 Risk Engine; T1D type 1 diabetes mellitus.

Logistic regression models were constructed to examine the factors involved in progression (dependent variable); all the models were adjusted by follow-up time between explorations. In model 1, including all the variables included in ST1RE separately, only being older (per 5 years) (OR [95% CI]: 1.38 [1.07–1.77], *p* = 0.015) and being an active smoker (OR [95% CI]: 3.29 [1.44–7.49], *p* = 0.005) were independently associated with progression of carotid atherosclerosis (Figure 2A).

Considering the result of the ST1RE score, there was a stepped association between the score and the risk of progression compared to those with low risk: (OR [95% CI]: 3.27 [1.12–9.53], *p* = 0.03) for medium and (OR [95% CI]: 5.85 [1.90–17.99], *p* = 0.002) for high-risk. (Figure 2B)

When adding other CVRFs in addition to ST1RE (model 3), ST1RE remained independently associated with the risk of progression (OR [95% CI]: 1.09 [1.03–1.15], *p* = 0.003) (Figure 2C). Similar results were observed if more advanced markers of atherosclerosis were assessed (presence of ≥2 plaques and ≥3 plaques; *p* < 0.05 for all models).

In an exploratory analysis in women, which included preeclampsia in addition to the cardiovascular risk factors in model 3, only ST1RE (OR [95% CI]: 1.09 [1.02–1.17]) and preeclampsia (OR [95% CI]: 3.79 [1.09–13.24]) remained independently associated with the risk of progression (*p* < 0.05).

### 3.3. Changes in Cardiovascular Risk Factors During the Follow-Up and Association with Atherosclerosis Progression

Following the initial comprehensive evaluation of CVR, there was a notable improvement in the management of CVRFs during the follow-up period. This was reflected in lower HbA1c levels, reduced LDL-C levels, an increased proportion of participants receiving statin therapy, and a decrease in the number of active smokers. However, there was a higher CVR according to ST1RE, a higher proportion of hypertension with no changes in SBP, and a higher BMI; see Table 2.

We also assessed the achievement of CVRF targets according to current clinical guidelines. Overall, there was a significant improvement in CVRF management, with the proportion of patients achieving good metabolic control doubling and the proportion achieving LDL-C targets according to their atherosclerosis burden tripling. Additionally, the proportion of non-smokers increased. When considering the number of CVRFs under control, there was substantial improvement (see Table 2; *p* < 0.001).

**Figure 3:**
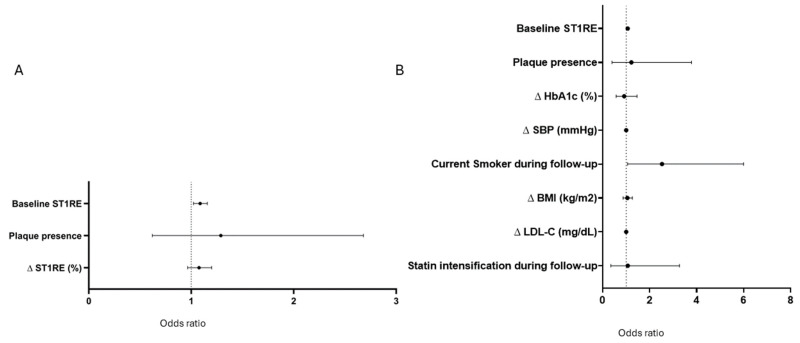
Logistic regression models with factors associated with the progression of atherosclerosis considering changes during follow-up. (**A**): With the ST1RE score plus the ST1RE score change during follow-up. (**B**): With the ST1RE score plus a change in other cardiovascular risk factors during follow-up. Abbreviations: BMI: body mass index; C: cholesterol; SBP: systolic blood pressure; ST1RE: Steno Type 1 Risk Engine.

In logistic regression models considering changes in ST1RE during follow-up and plaque presence, only the initial ST1RE was associated with plaque progression (OR [95% CI]: 1.09 [1.02–1.16]) (Figure 3A). With the addition of a change in CVRF (BMI, LDL-C, SBP, HbA1c, and smoking status), only the initial ST1RE (OR [95% CI]: 1.06 [1.02–1.11]) and being an active smoker during follow-up (OR [95% CI]: 2.52 [1.06–5.99]) remained independently associated with progression (Figure 3B).

When evaluating changes in the CVRF according to the initial ST1RE category, participants in the high-risk ST1RE category showed the greatest improvements in CVRFs and target attainment over time, despite all ST1RE groups demonstrating some degree of improvement (Supplementary Table S1).

At baseline, individuals with higher plaque burden had more hypertension and worse LDL-C levels, resulting in higher ST1RE scores. During follow-up, statin use increased, and LDL-C improved in this group, though hypertension and ST1RE scores remained elevated, especially in those with advanced atherosclerosis. Smoking cessation was observed only among individuals without atherosclerosis (Supplementary Table S2).

When stratified by progression status, both progressors and non-progressors showed similar CVRF trends; however, progressors consistently had higher ST1RE scores and greater tobacco use (Supplementary Table S3).

Lastly, we evaluated the changes and target attainment of CVRF according to sex (Supplementary Table S4). Although women generally exhibited lower CVR, they were more frequently smokers and less likely to achieve LDL-C target levels compared to men.

## 4. Discussion

In this prospective study including 151 individuals with T1DM in primary prevention, we found that atherosclerosis is prevalent, affecting almost half of the cohort. Over a follow-up period exceeding five years, 40% of participants showed progression in the number of carotid plaques, regardless of their initial atherosclerotic burden. The strongest predictors of atherosclerosis progression were age and tobacco use. However, the most significant factor associated with progression after adjusting for other CVRFs was the ST1RE score. The use of ST1RE could be instrumental in identifying individuals at higher risk for atherosclerosis exacerbation. To our knowledge, this is the first longitudinal study to evaluate the trajectories of carotid subclinical atherosclerosis and its determinants of progression.

Few studies in the general population have explored factors linked to plaque progression. The PESA study reported progression in one-third of non-diabetic participants over six years, with male sex, older age, and smoking as key predictors [14]. Consistent with these findings, our T1DM cohort also showed associations between progression, age, and smoking. However, the rate of progression was higher (40%) in our population, despite greater statin use and lower LDL-C levels compared to PESA. Notably, unlike in the general population, where progression is typically tied to baseline atherosclerosis [28], progression in our cohort occurred regardless of initial plaque burden. These findings highlight the accelerated atherosclerotic process in individuals with T1DM, likely driven by chronic metabolic dysfunction, despite intensive cardiovascular risk management [1,29].

In this study, we employed a pragmatic approach to define subclinical atherosclerosis progression, focusing on the increase in the number of carotid plaques. Previous studies have assessed subclinical carotid atherosclerosis progression in T1DM using IMT progression, yielding inconclusive results [30]. In contrast, the presence of carotid plaque has been strongly correlated with cardiovascular events in the general population [11,12]. Notably, Sojo-Vega et al. [10] found a relationship between basal subclinical atherosclerosis and increased mortality in T1DM, despite a relatively low incidence of cardiovascular events.

Previous studies have examined coronary artery calcium (CAC) as a surrogate for subclinical atherosclerosis in T1DM, with progression associated with BMI, non-HDL-C, ACR, and reduced eGFR [31,32,33]. In contrast, our cohort showed no significant association between DKD-related factors and atherosclerosis progression, despite similar DKD prevalence and renal indices. This discrepancy may be explained by differences in age, glycemic control, and higher statin use in our population. Unlike CAC, which reflects more advanced, calcified lesions and may be confounded by statin use [34], carotid plaque assessment captures earlier, non-calcified changes and may offer a more sensitive marker of atherosclerosis progression in T1DM [14,28].

Consistent with recent studies reporting no association between carotid intima-media thickness (CIMT) and long-term glycemic control [35], our findings showed no differences in HbA1c, hypoglycemia unawareness, or severe hypoglycemia between individuals with and without atherosclerosis progression.

In T1DM, cardiovascular risk remains elevated despite well-controlled traditional risk factors [4]. Our study incorporated carotid ultrasound within a comprehensive cardiovascular assessment, aligning with holistic approaches recommended in the literature [17,36]. The ST1RE, validated for predicting cardiovascular events and preclinical atherosclerosis in T1DM [16,21], was the only variable that remained significantly associated with plaque progression when accounting for multiple risk factors. A clear stepwise increase in progression risk corresponded with higher ST1RE scores, consistent with prior research [16,37]. Although most CVRFs improved over time, systolic blood pressure and BMI did not, and overall cardiovascular risk remained elevated, likely influenced by age—a key component of the ST1RE [21]. Participants with higher baseline ST1RE scores showed greater improvements except for hypertension. These findings support regular cardiovascular risk assessment using ST1RE in clinical practice to guide personalized risk reduction strategies in T1DM. The baseline ST1RE score was found to be independently associated with disease progression, even when accounting for changes in CVRFs. Smoking was the ST1RE component most strongly associated with progression risk, as those who progressed were more frequently smokers and had higher CTC, consistent with recent studies in T1DM [15].

In T1DM, no standardized tool exists to predict long-term cardiovascular event risk. T1DM-specific models outperform those for type 2 diabetes or the general population [38], with the ST1RE and the Scottish–Swedish score showing good discrimination and calibration even in ethnically diverse cohorts [39]. We selected ST1RE for its strong predictive accuracy, correlation with atherosclerotic burden [37], superiority over ESC guideline-based estimates [16], and broad applicability. Although new lifetime CVR models are emerging [40,41], further validation is needed before routine use. Incorporating validated CVR scores into clinical care may enable earlier identification of high-risk individuals and more effective preventive strategies to reduce cardiovascular event incidence.

In an exploratory analysis of women, preeclampsia, alongside ST1RE, was linked to increased odds of atherosclerosis progression. Preeclampsia is recognized as a risk-enhancing factor and has been associated with atherosclerosis in T1DM [42,43]. Sex differences remain a critical issue, with women often underdiagnosed and undertreated. In T1DM, women experience a greater reduction in life expectancy compared to men [2,44]. Despite lower ST1RE scores, women in our cohort exhibited poorer CVRF control, notably higher smoking rates, and elevated LDL-C. Further research is needed to investigate gender disparities and barriers to optimal care in this population.

This study has several strengths and limitations. The primary strength is that, to the best of our knowledge, it is the first study in individuals with T1DM to explore factors involved in the progression of subclinical atherosclerosis. Additionally, it evaluated changes in CVRFs within a specialized cardiovascular assessment program in a dedicated diabetes unit. The study also benefits from prospective, detailed data collection within a specialized cardiovascular program and adjustment for key confounders, enhancing result validity. However, there are some limitations. First, our cohort was derived exclusively from a single tertiary medical center, which may limit generalizability to other populations and settings. Second, during the study period, new guidelines on hypertension were introduced [25], and the criteria for treatment goals were updated. As a result, conclusions regarding the hypertension goal of <130/80 mmHg should be interpreted with caution. More precise BP assessments, such as ambulatory BP monitoring, should be considered in future studies to enhance accuracy. Third, we included a high-risk population with T1DM, selected according to statin treatment eligibility criteria defined in current international guidelines. This approach allowed us to focus on individuals most likely to benefit from preventive interventions and to detect measurable subclinical atherosclerotic burden; however, it limits the applicability of our findings to younger or lower-risk individuals with T1DM. Finally, the definition of atherosclerosis progression used in this study is not yet standardized and may not be optimal. Other definitions could potentially yield different progression rates.

In summary, despite advances and improvements in diabetes care, we found that atherosclerosis progression is very common in T1DM regardless of initial atherosclerosis burden. As the ST1RE score is independently associated with this progression, this tool could aid in identifying individuals at high risk of atherosclerosis exacerbation and, consequently, future cardiovascular events.

## Figures and Tables

**Table 1:** Characteristics of the study participants according to progression status.

	All N = 151	Non-Progressors N = 90	Progressors N = 61	*p* Value
Clinical characteristics				
Age (years)	49.81 ± 8.94	48.02 ± 9.04	52.47 ± 8.16	0.002
Sex (Female)	84 (55.6)	51 (56.7)	33 (54.1)	0.755
T1DM duration (years)	27.73 (22.01–32.35)	24.71 (19.95–32.33)	27.44 (23.33–32.57)	0.102
Dyslipidemia	80 (53.0)	47 (52.2)	33 (54.1)	0.821
Hypertension	42 (27.8)	25 (27.8)	17 (27.9)	0.990
Active smoking	41 (27.2)	18 (20.0)	23 (37.7)	0.016
CTC (packs/year)	4 (0–15)	0 (0–10)	10 (0–20)	0.005
Never smoker	68 (45.0)	48 (53.3)	20 (32.8)	0.013
Central obesity	44 (29.1)	26 (36.1)	18 (43.9)	0.414
BMI (kg/m^2^)	26.55 ± 4.05	26.55 ± 4.04	26.55 ± 4.09	0.997
CVD family history *	16 (10.6)	8 (8.9)	8 (13.1)	0.408
Microvascular complications	72 (47.7)	43 (47.8)	29 (47.5)	0.977
Diabetic kidney disease	15 (9.9)	10 (11.1)	5 (8.2)	0.557
Retinopathy	64 (42.4)	39 (43.3)	25 (41.0)	0.774
CSII therapy	62 (41.1)	40 (44.4)	22 (36.1)	0.304
Preeclampsia (in women)	19 (22.6)	7 (13.7)	12 (36.4)	0.051
Severe hypoglycemia	28 (18.5)	19 (21.1)	9 (14.8)	0.324
Hypoglycemia unawareness	25 (16.6)	17 (18.9)	8 (13.1)	0.349
ST1RE	15.92 (11.27–23.27)	15.03 (9.62–19.92)	17.60 (12.88–25.35)	0.006
Laboratory characteristics				
Serum Creatinine (mg/dL)	0.83 (0.71–0.96)	0.81 (0.71–0.97)	0.86 (0.74–0.95)	0.455
eGFR (CKD-EPI; ml/min/1.73 m^2^)	94.47 (80.26–103.98)	96.35 (82.32–105.95)	92.03 (77.79–102.69)	0.078
Albumin to creatinine ratio (mg/g)	4 (2–10)	4 (2–11)	4 (3–6.5)	0.140
HbA1c (mmol/mol, mean last 2 years)	58.6 ± 8.5	58.5 ± 8.9	58.7 ± 8.1	0.907
HbA1c (%, mean last 2 years)	7.51 ± 0.78	7.50 ± 0.81	7.52 ± 0.74	0.907
Total cholesterol (mg/dL)	192.07 ± 28.82	193.50 ± 28.46	189.95 ± 29.44	0.460
Triglycerides (mg/dL)	73 (59–101)	72 (61–95.75)	74 (57.5–103)	0.888
HDL-C (mg/dL)	62.71 ± 15.30	61.91 ± 13.88	63.88 ± 17.25	0.440
LDL-C (mg/dL)	112.76 ± 24.32	114.93 ± 24.60	109.56 ± 23.74	0.183
Lipoprotein (a) (mg/dL)	15 (9–38.5)	16 (9–60)	12 (9–29)	0.110
Non HDL-C (mg/dL)	129 ± 26.20	131.59 ± 26.92	126.07 ± 24.98	0.206
eGDR (mg/kg/min)	8.82 (6.85–10.03)	8.86 (6.92–10.03)	8.76 (6.80–10.20)	0.908
Pharmacological treatment				
LLT	73 (48.3)	43 (47.8)	30 (49.2)	0.866
ACEi/ARB	47 (31.1)	29 (32.2)	18 (29.5)	0.724
Antiplatelet drugs	12 (7.9)	6 (6.7)	6 (9.8)	0.480
Carotid US				
Plaque presence	64 (42.4)	33 (36.7)	31 (50.8)	0.084
≥2 plaques	40 (26.5)	18 (20)	22 (36.1)	0.028
≥3 plaques	21 (13.9)	8 (8.9)	13 (21.3)	0.030

Data are shown as *n* (percentage in each column), mean ± SD, or median (P25-75). ACEi: angiotensin-converting enzyme inhibitor; ARB: angiotensin receptor blocker; BMI: body mass index; C: cholesterol; CTC: Cumulative tobacco consumption; CVD: cardiovascular disease; CSII: continuous subcutaneous insulin infusion; eGDR: estimated glucose disposal rate; eGFR: estimated glomerular filtration rate; LLT: lipid-lowering therapy; ST1RE: Steno Type 1 Risk Engine; T1DM: type 1 diabetes mellitus; US: ultrasound. * Defined as <55 years in men and <65 years in women.

**Table 2:** Changes in CVRF and treatment during follow-up.

N = 151	Initial	Final	*p* Value
HbA1c (%)	7.51 ± 0.78	7.26 ± 0.78	<0.001
Statin treatment	73 (48.3)	104 (68.9)	<0.001
LDL-cholesterol (mg/dL)	112.76 ± 24.32	94.05 ± 29.55	<0.001
Hypertension	42 (27.8)	52 (34.4)	0.021
SBP (mmHg)	128.17 ± 15.35	130.13 ± 14.70	0.052
BMI (kg/m^2^)	26.55 ± 4.05	26.90 ± 4.90	0.015
Current smoker	41 (27.2)	32 (21.2)	0.022
ST1RE (%)	17.35 ± 8.81	21.16 ± 10.10	<0.001
Consecution of CVRF goals			
HbA1c < 53 mmol/mol (<7%)	27 (17.9)	50 (33.1)	<0.001
BP < 130/80 mmHg	37 (24.5)	37 (24.5)	0.095
LDL-C according to plaques	24 (15.9)	67 (44.4)	<0.001
Noncurrent smoker	110 (72.8)	119 (78.8)	0.013
No CVRF at goals	17 (11.3)	11 (7.3)	<0.001
1 CVRF at goal	78 (52.0)	43 (28.5)	
2 CVRFs at goals	46 (30.7)	57 (37.7)	
3 CVRFs at goals	9 (6.0)	35 (23.2)	
4 CVRFs at goals	0 (0)	5 (3.3)	

The data are presented as mean ± standard deviation or as number (percentage). BP: blood pressure; C: cholesterol; CVRF: cardiovascular risk factor; SBP: systolic blood pressure; ST1RE: Steno Type 1 Risk Engine.

## Data Availability

The data supporting the findings of this study are not publicly available due to privacy and ethical restrictions, as they include sensitive clinical and personal health information from participants. However, the data can be made available from the corresponding author upon reasonable request, provided that appropriate ethical approvals and data protection regulations are respected.

## Supplementary Materials

The following supporting information can be downloaded at: https://www.mdpi.com/article/10.3390/jcm14176004/s1 Factors Involved in the Progression of Preclinical Atherosclerosis in People with Type 1 Diabetes.
