# Supplementary material for: Factors Involved in the Progression of Preclinical Atherosclerosis in People with Type 1 Diabetes
Source: J Clin Med. 2025 Aug 25;14(17):6004. doi: 10.3390/jcm14176004 (PMC12429634; doi:10.3390/jcm14176004)
Supplement: Supplementary file 1 [file jcm-14-06004-s001.zip › jcm-3810378-supplementary.pdf]

SUPLEMENTARY MATERIAL

Title: **FACTORS INVOLVED IN THE PROGRESSION OF PRECLINICAL ATHEROSCLEROSIS IN PEOPLE WITH TYPE 1 DIABETES**

Table S1: Changes in CVRF during follow-up and consecution of CVRF goals according to initial steno type 1 risk engine (ST1RE).

	Low risk (n=29)			Medium risk (n=74)			High risk (n=48)				
	Initial	Final	P Value	Initial	Final	P Value	Initial	Final	P Value	P Initial between groups	P Final between groups
HbA1c (%)	7.42±0.93	7.03±0.84	0.029	7.48±0.76	7.39±0.68	0.267	7.60±0.78	7.21±0.85	<0.001	0.578	0.086
Statin treatment	9 (31.0)	12 (41.4)	0.375	36 (48.6)	52 (70.3)	<0.001	28 (58.3)	40 (83.3)	<0.001	0.067	<0.001
LDL cholesterol (mg/dl)	119.34±25.89	107.17±29.87	0.003	110.59±20.08	96.47±28.24	<0.001	112.13±28.77	94.05±29.55	<0.001	0.255	<0.001
Hypertension	2 (6.9)	4 (13.8)	0.500	17 (23)	22 (29.7)	0.180	23 (47.9)	26 (54.2)	0.375	<0.001	<0.001
SBP (mmHg)	118.48±13.71	123.07±13.88	0.069	126.41±14.62	128.90±14.29	0.155	137.04±12.83	136.26±13.64	0.680	<0.001	<0.001
BMI (kg/m^2)	25.85±4.02	26.54±5.29	0.106	26.73±4.43	27.01±5.00	0.120	26.69±3.45	26.88±4.89	0.558	0.584	0.909
Current smoker	7 (24.1)	5 (17.2)	0.500	20 (27.0)	19 (25.7)	1.00	14 (29.2)	8 (16.7)	0.031	0.890	0.417
ST1RE (%)	6.53±2.12	9.64±3.19	<0.001	14.72±2.70	19.60±5.46	<0.001	27.88±5.99	30.47±9.88	0.015	<0.001	<0.001
Consecution of CVRF goals											
HbA1c < 53 mmol/mol (< 7%)	8 (27.6)	14 (48.3)	0.058	12 (16.2)	19 (25.7)	0.090	7 (14.6)	17 (35.4)	0.008	0.308	0.083
BP<130/80 mmHg	11 (37.9)	10 (34.5)	0.705	20 (27.4)	24 (32.4)	0.285	6 (12.5)	12 (25.0)	0.034	0.032	0.597

	Low risk (n=29)			Medium risk (n=74)			High risk (n=48)				
	Initial	*Final*	*P Value*	*Initial*	*Final*	*P Value*	*Initial*	*Final*	*P Value*	*P Initial between groups*	*P Final between groups*
LDL-C according to plaques	6 (20.7)	14 (48.3)	0.011	13 (17.6)	28 (37.8)	0.002	5 (10.4)	25 (52.1)	<0.001	0.421	0.270
Noncurrent smoker	22 (75.9)	24 (82.8)	0.157	54 (73.0)	55 (74.3)	0.655	34 (70.8)	40 (83.3)	0.014	0.890	0.417
None CVRF	3 (10.3)	1 (3.4)	0.005	7 (9.6)	7 (9.5)	0.004	7 (14.6)	3 (6.3)	<0.001	0.043	0.274
1 CVRF	9 (31)	7 (24.1)		38 (52.1)	25 (33.8)		31 (61.6)	11 (22.9)			
2 CVRF	13 (44.8)	10 (34.5)		24 (32.9)	28 (37.8)		9 (18.8)	19 (39.6)			
3 CVRF	4 (13.8)	9 (31.0)		4 (5.5)	11 (14.9)		1 (2.1)	15 (31.3)			
4 CVRF	0 (0)	2 (6.9)		0 (0)	3 (4.1)		0 (0)	15 (31.3)			

BMI: body mass index; BP: blood pressure; CVRF: cardiovascular risk factor; LDL-C: low density lipoprotein cholesterol; ST1RE: steno type 1 risk engine; SBP systolic blood pressure.

The data are presented as mean ± standard deviation or as number (percentage).

Table S2:

Changes in CVRF during follow-up and consecution of CVRF goals according to initial plaque presence.

	0 PLAQUE (n=87)			1-2 PLAQUES and <3 MM (n=42)			≥3 PLAQUES OR >3 MM (n=22)				
	Initial	*Final*	*P Value*	*Initial*	*Final*	*P Value*	*Initial*	*Final*	*P Value*	*P Initial between groups*	*P Final between groups*
HbA1c (%)	7.52±0.81	7.20±0.78	<0.001	7.43±0.70	7.40±0.73	0.382	7.61±0.81	7.25±0.87	0.007	0.805	0.451
Statin treatment	41 (47.1)	48 (55.2)	0.092	18 (42.8)	36 (85.7)	<0.001	14 (63.6)	20 (90.9)	0.031	0.327	<0.001
LDL cholesterol (mg/dl)	114.86±24.55	104.66±26.88	<0.001	112.40±24.20	87.86±26.29	<0.001	105.14±23.06	63.91±20.83	<0.001	0.110	<0.001
Hypertension	19 (21.8)	25 (28.7)	0.109	10 (23.8)	12 (28.6)	0.625	13 (59.1)	15 (68.2)	0.50	0.003	0.004
SBP (mmHg)	124.72±15.48	127.17±14.09	0.073	129.18±14.02	131.69±15.00	0.138	139.73±11.32	138.70±13.13	0.333	<0.001	<0.001
BMI (kg/m²)	26.77±4.10	27.35±4.97	0.003	25.74±4.07	25.74±4.80	0.495	27.17±3.74	27.26±4.66	0.399	0.870	0.457
Current smoker	23 (26.4)	15 (17.2)	0.008	13 (31)	12 (28.6)	1.000	5 (22.7)	5 (22.7)	1.000	0.930	0.306
ST1RE (%)	14.36±7.84	17.87±9.01	<0.001	18.56±6.57	22.70±8.29	<0.001	26.74±9.23	31.05±10.50	<0.001	<0.001	<0.001
Consecution of CVRF goals											
HbA1c < 53 mmol/mol (< 7%)	17 (19.5)	29 (33.3)	0.019	6 (14.3)	12 (28.6)	0.058	4 (18.2)	9 (40.9)	0.025	0.691	0.720

	0 PLAQUE (n=87)			*1-2 PLAQUES and <3 MM (n=42)*			*≥3 PLAQUES OR >3 MM (n=22)*				
	Initial	*Final*	*P Value*	*Initial*	*Final*	*P Value*	*Initial*	*Final*	*P Value*	*P Initial between groups*	*P Final between groups*
BP<130/80 mmHg	23 (26.7)	24 (27.6)	0.808	11 (26.2)	18 (42.9)	0.020	3 (13.6)	4 (18.2)	0.564	0.278	0.962
LDL-C according to plaques	22 (25.3)	48 (55.2)	<0.001	2 (4.8)	10 (23.8)	0.021	0 (0)	9 (40.9)	<0.001	<0.001	0.024
Noncurrent smoker	64 (73.6)	72 (82.8)	0.005	29 (69)	30 (71.4)	0.655	17 (77.3)	17 (77.3)	1.000	0.930	0.306
None CVRF	9 (10.5)	6 (6.9)	0.317	6 (14.3)	4 (9.5)	0.005	2 (9.1)	1 (4.5)	0.317	0.012	0.146
1 CVRF	37 (43)	19 (21.8)		25 (59.5)	15 (35.7)		16 (72.7)	9 (40.9)			
2 CVRF	32 (37.2)	37 (42.5)		10 (23.8)	14 (33.3)		4 (18.2)	6 (27.3)			
3 CVRF	8 (9.3)	20 (23)		1 (2.4)	9 (21.4)		0 (0)	6 (27.3)			
4 CVRF	0 (0)	5 (5.7)		0 (0)	0 (0)		0 (0)	0 (0)			

BMI: body mass index; BP: blood pressure; CVRF: cardiovascular risk factor; LDL-C: low density lipoprotein cholesterol; ST1RE: steno type 1 risk engine; SBP systolic blood pressure.

The data are presented as mean ± standard deviation or as number (percentage).

Table S3:

Changes in CVRF during follow-up and consecution of CVRF goals according to progression status.

	Non progressors (n=90)			Progressors (n=61)				
	Initial	Final	P Value	Initial	Final	P Value	P Initial between groups	P Final between groups
HbA1c (%)	7.50±0.81	7.28±0.81	0.009	7.52±0.74	7.23±0.73	<0.001	0.472	0.348
Statin treatment	43 (47.8)	60 (66.7)	<0.001	30 (49.2)	44 (72.1)	<0.001	0.866	0.477
LDL cholesterol (mg/dl)	114.93±24.60	97.34±31.21	<0.001	109.56±23.74	89.19±26.41	<0.001	0.092	0.048
Hypertension	25 (27.8)	27 (30)	0.688	17 (27.9)	25 (41)	<0.021	0.990	0.163
SBP (mmHg)	127.05±15.52	128.52±14.80	0.161	129.85±15.06	132.55±14.32	0.095	0.119	0.050
BMI (kg/m^2)	26.55±4.04	26.89±5.01	0.064	26.55±4.09	26.90±4.78	0.055	0.499	0.478
Current smoker	18 (20)	14 (15.6)	0.125	23 (37.7)	18 (29.5)	0.180	0.016	0.040
ST1RE (%)	15.62±8.17	18.99±9.61	<0.001	19.96±9.14	24.39±10.03	<0.001	0.002	<0.001
Consecution of CVRF goals								
HbA1c < 53 mmol/mol (< 7%)	17 (18.9)	25 (27.8)	0.102	10 (16.4)	25 (41)	<0.001	0.695	0.091
BP<130/80 mmHg	25 (27.8)	27 (30)	0.637	12 (20)	19 (31.1)	0.035	0.279	0.880
LDL-C according to plaques	13 (14.4)	41 (45.6)	<0.001	11 (18)	26 (42.6)	0.002	0.554	0.722
Noncurrent smoker	72 (80)	76 (84.4)	0.046	38 (62.3)	43 (70.5)	0.096	0.016	0.040
None CVRF	8 (8.9)	4 (4.4)	0.059	9 (15)	7 (11.5)	0.564	0.280	0.421
1 CVRF	44 (48.9)	28 (31.1)		34 (56.7)	15 (24.6)			
2 CVRF	31 (34.4)	35 (38.9)		15 (25)	22 (36.1)			
3 CVRF	7 (7.8)	21 (23.3)		2 (3.3)	14 (23)			
4 CVRF	0 (0)	2 (2.2)		0 (0)	3 (4.9)			

BMI: body mass index; BP: blood pressure; CVRF: cardiovascular risk factor; LDL-C: low density lipoprotein cholesterol; ST1RE: steno type 1 risk engine; SBP systolic blood pressure.

The data are presented as mean ± standard deviation or as number (percentage).

Table S4:

Changes in CVRF during follow-up and consecution of CVRF goals according to sex.

	Women (n=84)			Men (n=67)				
	Initial	Final	P Value	Initial	Final	P Value	P Initial between groups	P Final between groups
HbA1c (%)	7.56±0.81	7.31±0.84	0.013	7.44±0.74	7.20±0.70	0.003	0.174	0.194
Statin treatment	41 (48.8)	56 (66.7)	<0.001	32 (47.8)	48 (71.6)	<0.001	0.898	0.512
LDL cholesterol (mg/dl)	114.11±21.95	98.20±27.97	<0.001	111.07±27.08	88.86±30.84	<0.001	0.224	0.027
Hypertension	20 (23.8)	25 (29.8)	0.18	22 (32.8)	27 (40.3)	0.125	0.219	0.176
SBP (mmHg)	126.33±16.19	127.33±14.37	0.476	130.69±13.97	133.59±14.47	0.095	0.042	0.005
BMI (kg/m^2)	26.14±4.41	26.40±5.37	0.242	27.06±3.51	27.48±4.17	0.029	0.083	0.088
Current smoker	30 (35.7)	24 (28.6)	0.07	11 (16.4)	8 (11.9)	0.375	0.008	0.013
ST1RE (%)	16.30±9.04	19.75±10.15	<0.001	18.62±8.33	22.90±9.83	<0.001	0.053	0.028
Consecution of CVRF goals								
HbA1c < 53 mmol/mol (< 7%)	14 (16.7)	25 (29.8)	0.016	13 (19.4)	25 (37.3)	0.007	0.663	0.327
BP<130/80 mmHg	26 (31.3)	27 (32.1)	0.808	11 (16.4)	19 (28.4)	0.021	0.035	0.616
LDL-C according to plaques	14 (16.7)	31 (36.9)	<0.001	10 (14.9)	36 (53.7)	<0.001	0.771	0.039
Noncurrent smoker	54 (64.3)	60 (71.4)	0.034	56 (83.6)	59 (88.1)	0.180	0.008	0.013
None CVRF	15 (18.1)	9 (10.7)	<0.001	2 (3)	2 (3)	<0.001	0.009	0.147
1 CVRF	35 (42.2)	28 (33.3)		43 (64.2)	15 (22.4)			
2 CVRF	27 (32.5)	28 (33.3)		19 (28.4)	29 (43.3)			
3 CVRF	6 (7.2)	17 (20.2)		3 (4.5)	18 (26.9)			
4 CVRF	0 (0)	2 (2.4)		0 (0)	3 (4.5)			

BMI: body mass index; BP: blood pressure; CVRF: cardiovascular risk factor; LDL-C: low density lipoprotein cholesterol; ST1RE: steno type 1 risk engine; SBP systolic blood pressure.

The data are presented as mean ± standard deviation or as number (percentage).
